# Involvement of heparanase in the pathogenesis of acute pancreatitis: Implication of novel therapeutic approaches

**DOI:** 10.1111/jcmm.18512

**Published:** 2024-09-09

**Authors:** Dalit B. Hamo‐Giladi, Ahmad Fokra, Edmond Sabo, Aviva Kabala, Irena Minkov, Shadi Hamoud, Salim Hadad, Zaid Abassi, Iyad Khamaysi

**Affiliations:** ^1^ Department of Physiology The Ruth & Bruce Rappaport Faculty of Medicine Haifa Israel; ^2^ Department of Pathology Carmel Hospital Haifa Israel; ^3^ Department of Pathology Rambam Health Care Center Haifa Israel; ^4^ Department of Internal Medicine E Rambam Health Care Center Haifa Israel; ^5^ Department of Pharmacy Rambam Health Care Center Haifa Israel; ^6^ Department of Laboratory Medicine Rambam Health Care Center Haifa Israel; ^7^ Department of Gastroenterology Rambam Health Care Center Haifa Israel

**Keywords:** acute pancreatitis, amylase, heparanase, lipase, mitochondria, NSAIDs

## Abstract

Acute pancreatitis (AP) is a common gastrointestinal disease with high morbidity and mortality rate. Unfortunately, neither the etiology nor the pathophysiology of AP are fully understood and causal treatment options are not available. Recently we demonstrated that heparanase (Hpa) is adversely involved in the pathogenesis of AP and inhibition of this enzyme ameliorates the manifestation of the disease. Moreover, a pioneer study demonstrated that Aspirin has partial inhibitory effect on Hpa. Another compound, which possesses a mild pancreato‐protective effect against AP, is Trehalose, a common disaccharide. We hypothesized that combination of Aspirin, Trehalose, PG545 (Pixatimod) and SST0001 (Roneparstat), specific inhibitors of Hpa, may exert pancreato‐protective effect better than each drug alone. Thus, the current study examines the pancreato‐protective effects of Aspirin, Trehalose, PG545 and SST0001 in experimental model of AP induced by cerulein in wild‐type (WT) and Hpa over‐expressing (Hpa‐Tg) mice. Cerulein‐induced AP in WT mice was associated with significant rises in the serum levels of lipase (X4) and amylase (X3) with enhancement of pancreatic edema index, inflammatory response, and autophagy. Responses to cerulein were all more profound in Hpa‐Tg mice versus WT mice, evident by X7 and X5 folds increase in lipase and amylase levels, respectively. Treatment with Aspirin or Trehalose alone and even more so in combination with PG545 or SST0001 were highly effective, restoring the serum level of lipase back to the basal level. Importantly, a novel newly synthesized compound termed Aspirlose effectively ameliorated the pathogenesis of AP as a single agent. Collectively, the results strongly indicate that targeting Hpa by using anti‐Hpa drug combinations constitute a novel therapy for this common orphan disease.

## 1 INTRODUCTION

Acute pancreatitis (AP) is a common disease in gastroenterology with an increasing global incidence, accounting for about 3% of all hospitalized patients.^1^ AP is a complex inflammatory syndrome that results from many etiologies of which gallstones, alcohol and endoscopic retrograde cholangiopancreatography (ERCP) are the leading causes.^2^ About 20% of patients who experienced their first AP attack will develop recurrent attacks and approximately one‐third of the latter continue to end‐stage chronic pancreatitis.^3^, ^4^, ^5^ Despite the advances in medicine, the worldwide mortality rate among AP patients remained high, imposing an important burden on the healthcare system.^1^, ^2^ The relatively high morbidity and mortality characterizing AP could be attributed to the poor understanding of the pathogenesis of this common clinical setting. It is widely accepted that excessive stimulation of the pancreas or direct destructive insults obstruct the outflow of zymogen granules, where they are proteolytically activated in the acinar cells by lysosomal enzymes mainly cathepsin B and eventually causing acute cell injury.^6^ This adverse reaction is further exacerbated by neutrophilic enzymes and transcription factors, which lead to the production of various pro‐inflammatory cytokines, including tumour necrosis factor (TNF)‐α, interleukin (IL)‐1, IL‐6, and IL‐8, along with the conversion of trypsinogen into trypsin—a phenomenon also referred to as auto‐digestion.^7^, ^8^ Moreover, the pro‐inflammatory stimuli upregulate cyclooxygenase (COX)‐2, a key enzyme responsible for the generation of prostaglandins (PGs), leukotrienes, and thromboxane from arachidonic acid.^9^

In light of the unclear characterization of the mechanistic pathways responsible for AP, treatment options targeting a specific underlying cause remain elusive and the current therapy relies mainly on painkillers and hydration, opioids being the most frequently prescribed analgesics for pain relief of patients with AP.^10^, ^11^ As AP is secondary to pancreatic parenchymal inflammation, non‐steroidal anti‐inflammatory drugs (NSAIDs) are also often used.^10^, ^11^ Previous studies have highlighted the keen involvement of heparanase (Hpa), an endoglycosidase that degrades heparan sulphate (HS),^12^ in the pathogenesis of inflammatory diseases including AP.^12^, ^13^, ^14^, ^15^ Specifically, we provided evidence that pancreatic Hpa expression and activity are significantly increased following cerulein‐induced AP.^12^ Moreover, pancreas edema and inflammation, as well as the induction of cytokines and signalling molecules in response to cerulein were attenuated markedly by PG545 and SST0001, heparin/HS‐like Hpa inhibitors,^12^ implying that the enzyme plays a significant role in AP. Notably, the above features appear even more pronounced in transgenic mice overexpressing Hpa, suggesting that these mice can be utilized as a highly appropriate model system to reveal the molecular mechanism by which Hpa functions in AP.^12^ Recently, a pioneering study has demonstrated that Aspirin has partial inhibitory effect on Hpa,^16^ a feature that may be held responsible, in part, for the anti‐inflammatory effects of NSAIDs, including in AP^17^, ^18^ Another compound that possesses a potential pancreato‐protective effect against AP is Trehalose, a naturally occurring non‐reducing disaccharide.^19^ The mechanisms underlying the beneficial effect of Aspirin and Trehalose are unknown but assumingly involve Hpa inhibition and anti‐oxidative effect. The current study addresses the assumption that a combination of established Hpa inhibitors with Aspirin or Trehalose will ameliorate AP more efficiently than each drug alone.

## 2 MATERIALS AND METHODS

### 2.1 Animals

Studies utilized wild‐type (WT) BALB/c mice (*n* = 6–19) and heparanase transgenic (Hpa‐Tg) mice (*n* = 6–20) in which the human Hpa gene is driven by a constitutive β‐actin promoter^20^ in a BALB/c genetic background. Mice were fed standard mouse chow and tap water ad libitum. All experiments were approved and performed according to the Technion's guidelines of the Committee for the Supervision of Animal Experiments (IL‐90‐08‐2020).

### 2.2 Synthesis and characterization of aspirin‐trehalose conjugates

Acetylsalicylic Acid (Aspirin), D+ Trehalose dihydrate, dicyclohexylcarbodiimide (DCC), 4‐dimethylaminopyridine (DIMAP), hydroxybenzotriazole (HOBT), dimethyl foramide (DMF) and Reverse Phase C‐18 Silica Gel were purchased from Sigma Aldrich.

Spray for sugar detection: 1 mL of sulfuric acid dissolve in 10 mL of cold ethanol then 1 mL of anisaldehyde is added and stirred for 10 min.

In the existing literature, the synthesis of D+ Trehalose ester has been achieved through a multi‐step process involving five distinct steps. This method includes initial protection of primary and secondary alcohols using trityl chloride and benzyl chloride, respectively. Subsequently, the trityl group is removed, and the desired esterification is carried out with an aromatic acid derivative. This final esterification step employs DCC and DMAP as reagents, ultimately leading to the formation of ester.^21^ In this study, we present an alternative approach where the synthesis of Trehalose ester is achieved directly without the need for protective groups. This novel one‐step synthesis utilizes DCC, DMAP, and HOBT as key reagents. This study focuses on the synthesis and characterization of new conjugates by coupling Aspirin with Trehalose, a disaccharide. Various reaction conditions were explored to achieve successful synthesis, with a selected Trehalose‐to‐Aspirin ratio of 2:1. The resulting Mono and Di‐ester conjugates were purified and characterized using chromatographic techniques and spectroscopic analyses (Figure S1).

The synthesis of new chemical entity, specifically Aspirin–Trehalose conjugates, presents an innovative approach to potentially enhance the pharmacological effects of Aspirin–Trehalose conjugates (Aspirlose) while minimizing adverse effects.

1. Reaction components: Aspirin, DCC, DMAP, HOBT, and Trehalose were dissolved in DMF and stirred for 178 h in room temperature.
2. Purification: Precipitated dicyclohexylurea was removed by filtration, and the solvent was evaporated. The resulting ester conjugates were purified using reverse‐phase silica gel C‐18 chromatography column with varying solvent mixtures.
3. Characterization: The chemical structure was confirmed using LC–MS and NMR analyses.

Thin liquid chromatography (TLC) analysis was employed to detect the products containing the phenyl ring of Aspirin. The mobile phase consisted of various solvent ratios, allowing for visualization under UV light at 254 nm. As Trehalose does not absorb UV light, a 5% sulfuric acid solution in methanol was used to visualize Trehalose spots after heating.

The synthesis of Aspirin–Trehalose conjugates was successfully achieved using a Trehalose‐to‐Aspirin ratio of 2:1. The resulting mono and di‐ester conjugates were characterized using chromatography and spectroscopic techniques, confirming their chemical structure.

### 2.3 Induction of acute pancreatitis

Mice were injected with either cerulein (intraperitoneally, 50 mg/kg, five times at 1 h apart) (Sigma‐Aldrich), or saline (0.9% NaCl) (control group). Additional groups of mice were pre‐treated with PG545 (0.4 mg/mouse, i.p, 24–48 h prior to cerulein administration), SST0001 (1.0 mg/mouse, i.p, 24–48 h prior to the administration of cerulein), Trehalose (2 gr/kg, i.p.) Aspirin (250 mg/kg, ip) or Aspirlose (10 mg/kg, i.p.). PG545 and SST0001 were kindly provided by Zucero Therapeutics (Brisbane, Australia) and Leadiant Biosciences (Rome, Italy), respectively.^22^, ^23^ Mice were sacrificed 24 h later, and serum samples and pancreatic tissue were collected for measurements of blood amylase and lipase levels, pancreatic index (pancreas/body weight ratio), and for histological analysis. Portion of the pancreas tissue was homogenized and lysate samples were subjected to immunoblotting.

### 2.4 Pancreatic histopathological and ultrastructural analyses

#### 2.4.1 Light microscopy

Pancreatic tissues were removed from the various experiment groups, fixed in 10% neutral‐buffered formalin during 24 h, dehydrated with increased ethanol concentration, and then embedded in paraffin. 5‐μm tissue sections were deparaffinized, rehydrated and stained with haematoxylin and eosin (H&E). Briefly, Paraffin tissue sections was rehydrated under standard protocols, including clearing in xylene (three times), and rehydration in anhydrous alcohol (100% alcohol and 95% alcohol), and distilled water, successively. Five‐micron sections were stained with haematoxylin for 4 min, rinsed using tap water, and blotted dry. Next, slides were incubated with eosin stain for 4 min and followed by routine dehydration, including 95% alcohol and xylene. Finally, slides were sealed with a slide mounting medium–DPX.

#### 2.4.2 Electron microscopy

Pancreatic tissues from the various experimental groups were fixed in 3.5% glutaraldehyde and rinsed in 0.1 M sodium cacodylate buffer, pH 7.4. Tissue blocks (1 mm^3^) were post‐fixed with 2% OsO_4_ in 0.2 M cacodylate buffer for 1 h, rinsed again in cacodylate buffer to remove excess osmium, immersed in saturated aqueous uranyl acetate, dehydrated in graded alcohol solutions, immersed in propylene oxide, and embedded in Epon 812. Ultrathin sections (80 nm) were mounted on a 300‐mesh, thin‐bar copper grid, counterstained with saturated uranyl acetate and lead citrate. Sections were examined with a transmission electron microscope (E.M) (Jeol 1011 JEM), at 80 KV. In order to establish the cellular changes due to inflammatory response we applied morphometric analysis on E.M images by Image pro plus 7 software. Several images from each experimental group and from different mice were taken and all the mitochondria in every image were measured manually and analysed by software. One field considered as one cell (*n* = 3–11). The measurement was double blind, as each image marked by a number (for example, saline = 7) without a name. Later on, the software sorted the mitochondria measurement to the correct group. Different features of size, shape and texture of mitochondria measured and calculated for each sample. The analysis output revealed distinct features of healthy and AP condition and provided the visually and quantitative notion of mitochondrial swelling.

#### 2.4.3 Immunohistochemistry

Pancreatic tissues were removed from the various experimental groups, fixed in 10% neutral‐buffered formalin during 24 h, dehydrated with increased ethanol concentration, and then embedded in paraffin. Three micrometre tissue sections were deparaffinized, rehydrated and immunofluorescence staining for Hpa and F4/80 were performed. Briefly, following rehydration, antigen retrieval was performed using Proteinse K (cat# ab64220, Abcam). Sections were blocked for 60 min to block nonspecific binding with 10% Normal Donkey serum (cat# 017–000‐121, Jackson ImmunoResearch), then incubated either with anti‐Hpa (cat# ant‐155‐a, Prospec Protein Specialists, 1/100) or anti F4/80 (cat# ab16911, Abcam, 1/10) antibody overnight, 4°C. Next, sections were incubated with Cy™3 donkey anti‐rabbit secondary antibody (cat# 711–165‐152‐ Jackson ImmunoResearch, 1/100) or Cy™3 donkey anti‐rat secondary antibody (cat# 712‐165‐153‐ Jackson ImmunoResearch, 1/100). Finally, sections were mounted with DAPI Immunomount (Cat# 0100–20, SouthernBiotech), then were visualized using Zeiss Axio observer inverted microscope system (Zeiss Axio, 37030, Göttingen, Germany).

#### 2.4.4 Immunoblotting

Pancreatic tissue samples from the various experimental groups were homogenized on ice with lysis buffer and protein was quantified using Bradford commercial assay. Protein samples (50 μg for WT mice and 25 μg for Hpa‐Tg mice) were electrophoresed on sodium dodecyl sulphate (SDS) polyacrylamide gel (10%) under denaturing conditions, and then electro‐transferred to nitrocellulose membranes for 1.5 h at 100 V. Membranes were blocked with 5% bovine serum albumin (BSA) in Tris‐buffered saline (TBS) for 1 h at room temperature. Membranes subjected to anti‐Hpa (cat# ant‐155‐a, Prospec Protein Specialists, 1/1000), anti‐cathepsin L (CathL) (cat# sc‐6498, Santacruz Biotechnology, 1/1000), anti‐phospho‐STAT3 (p‐STAT3) (cat# sc‐8059, Santacruz Biotechnology, 1/500), anti‐TNFα (cat# ab6671, Abcam, 1/2000), anti‐LC3 (cat# L8918, Sigma‐Aldrich, 1/1000). Main antibodies were used with 5% BSA overnight at 4°C.

In order to serve as internal control, immuno‐detection of glyceraldehyde‐3‐phosphate dehydrogenase (GAPDH) with monoclonal anti‐GAPDH antibody (cat# sc‐32,233, Santacruz Biotechnology, 1/1000) was tested. HRP‐secondary antibodies were applied for 45 min at room temperature at a concentration of 1:10,000. The signal was detected with ECL (chemiluminescence substrate), and images were captured with Fusion FX7 Edge Spectra.

### 2.5 Quantitative real‐time PCR

Complete ribonucleic acid (RNA) was purified from fresh pancreatic tissues using a TRIzol reagent. The pancreas was removed quickly by carefully cutting it from the spleen and the small intestine. The pancreas was put in a tube containing cold ice TRIzol, homogenized for 5 s, and transferred to a new 2 mL microcentrifuge tube. The lysate was centrifuged at 4°C, 12000 g for 5 min, 1 mL of supernatant was transferred to a fresh 2 mL tube, 200 μL of chloroform was added, the tube was shaken vigorously for 15 s and the tube was centrifuged at 4°C, 12,000 g for 15 min. The aqueous phase was then transferred to a fresh tube, 0.5 mL of isopropranolol was added, and the tube was centrifuged at 4°C, 12,000 *g* for 10 min, then the RNA precipitate was washed with 1 mL of 75% ethanol, and finally the RNA pellet was briefly dried and eluted in 200 μL of nuclease‐free water (NFW) containing 1 μL Rnase inhibitor. RNA concentration was quantified by spectrophotometry using NanoDrop 2000, and RNA integrity was validated at the Genomics Core Facility in the Faculty of Medicine (Technion).^24^, ^25^ The complementary DNA (cDNA) was synthesized according to the manufacturer's protocol from complete RNA using the Maximal first strand cDNA synthesis kit for RT‐qPCR. Using PerfeCTa SYBR Green with the target gene primers, quantitative real‐time PCR analysis was performed and analysed in the 7500 Real Time PCR System (Applied Biosystems, RHENIUM 8440, USA). The mRNA levels of the various genes (Hpa, CathL, TNFα, IL‐6 and IL‐β) were standardized to mRNA levels of Rpl13a, referred as housekeeping gene. Relative to the normalized values obtained for saline group (control) at baseline, fold shift was measured. The following mouse primer sets were used:

1. Hpa: F‐CCAAGTGCTCGGGGTTAGAC, R‐AGAAACTGTTGGGCTCATTGC.
2. CathL: F‐CCCTATGAAGCGAAGGACGG, R‐CTGGAGAGACGGATGGCTTG.
3. TNF‐α: F‐CTATGTCTCAGCCTCTTCTC, R‐CATTTGGGAACTTCTCATCC.
4. IL‐6: F‐GTCTATACCACTTCACAAGTC, R‐TGCATCATCGTTGTTCATAC.
5. IL‐1β: F‐TGCCACCTTTTGACAGTGATG, R‐ATGTGCTGCTGCGAGATTTG.
6. Rpl13a: F‐AAGCAGGTACTTCTGGGCCG, R‐GGGGTTGGTATTCATCCGCT.

### 2.6 Statistical analysis

The results are shown as mean ± standard deviation (SD). Statistical significance was tested for comparisons between the various groups except of control subgroup (saline) using one‐way ANOVA followed by Bonferroni correction. *p* < 0.05 value is found statistically significant.

## 3 RESULTS

### 3.1 Amylase and lipase serum levels and pancreatic oedema index

**FIGURE 1:**
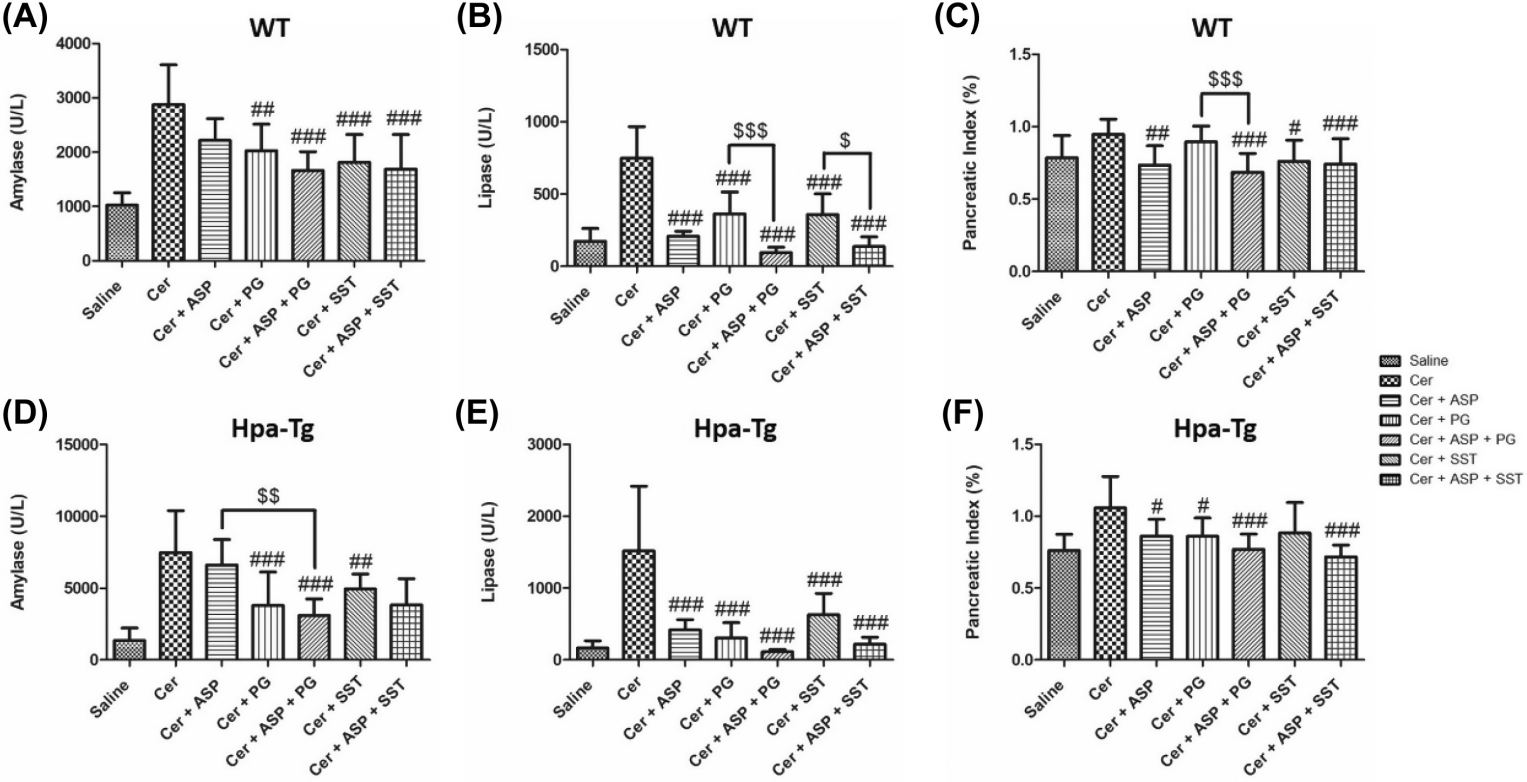
Effects of PG545, SST0001, Aspirin, or combined therapy on acute pancreatitis (AP). Serum levels of amylase (A, D), lipase (B, E) and pancreatic index (pancreas/body weight ratio) (C, F) in wild‐type (WT) and heparanase transgenic (Hpa‐Tg) mice subjected to cerulein‐induced AP. Blood samples were collected and evaluated biochemically for lipase and amylase levels from control untreated WT (saline, *n* = 15) or Hpa‐Tg (saline, *n* = 15) mice; Cerulein treated WT (cer, *n* = 15) or Hpa‐Tg (cer, *n* = 20) mice; Cerulein + Aspirin treated WT (cer + ASP, *n* = 10) or Hpa‐Tg (cer + ASP, *n* = 14) mice; Cerulein + PG545 treated WT (cer + PG, *n* = 15) or Hpa‐Tg (cer + PG, *n* = 15) mice; Cerulein + Aspirin + PG545 treated WT (cer + ASP + PG, *n* = 9) or Hpa‐Tg (cer + ASP + PG, *n* = 11) mice; Cerulein + SST0001 treated WT (cer + SST, *n* = 9) or Hpa‐Tg (cer + SST, *n* = 8) mice; Cerulein + Aspirin + SST0001 treated WT (cer + ASP + SST, *n* = 9) or Hpa‐Tg (cer + ASP + SST, *n* = 8) mice. Pancreases samples from the above mentioned groups were collected and weighted. #, *p* < 0.05; ##, *p* < 0.01; ###, *p* < 0.001 compared to cerulein group; $, *p* < 0.05; $$, *p* < 0.01; $$$, *p* < 0.001 compared to combination group.

**FIGURE 2:**
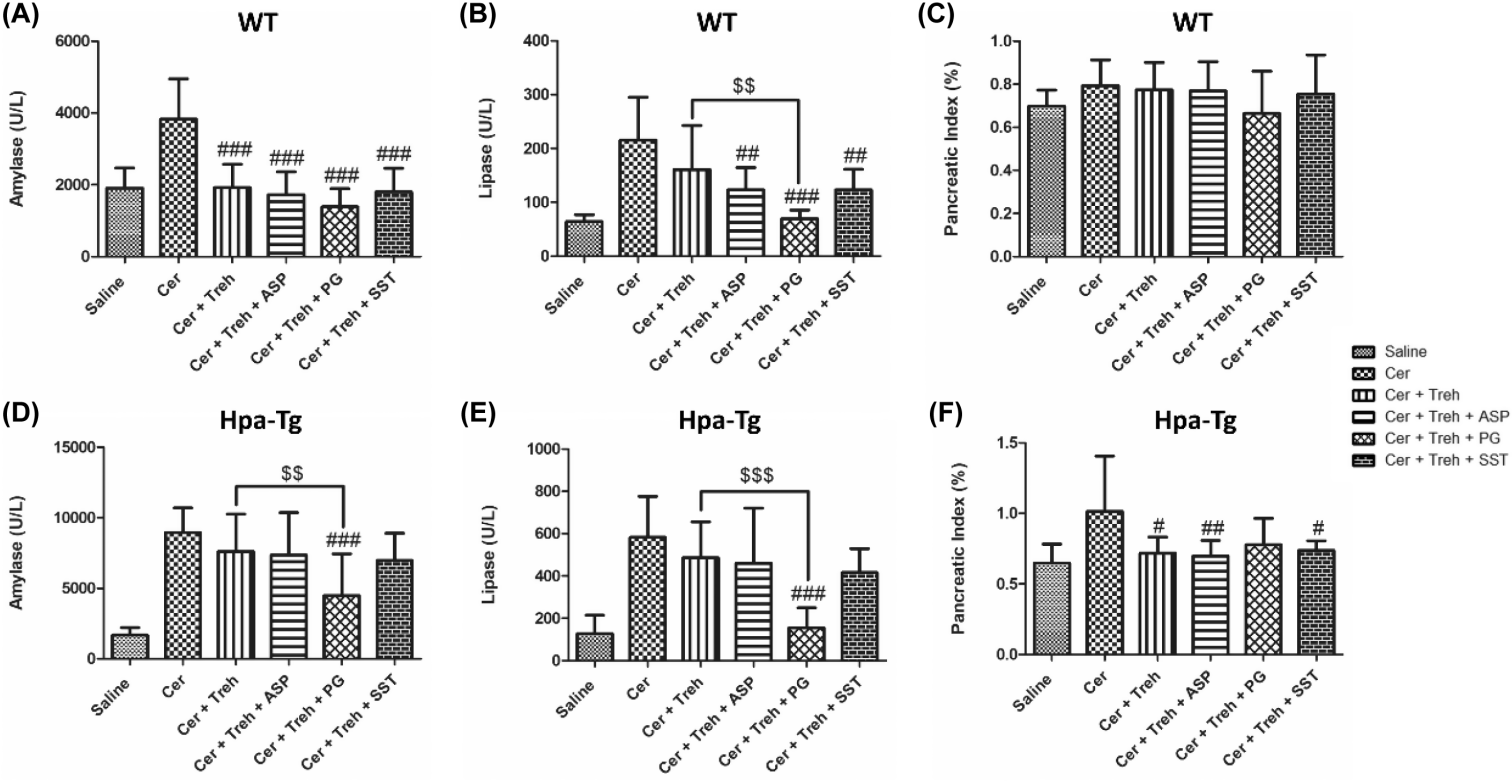
Effects of Trehalose, PG545, SST0001, Aspirin, or combined therapy on acute pancreatitis (AP). Serum levels of amylase (A, D), lipase (B, E) and pancreatic index (pancreas/body weight ratio) (C, F) in wild‐type (WT) and heparanase transgenic (Hpa‐Tg) mice subjected to cerulein‐induced AP. Blood samples were collected and evaluated biochemically for lipase and amylase levels from control untreated WT (saline, *n* = 15) or Hpa‐Tg (saline, *n* = 12) mice; Cerulein treated WT (cer, *n* = 19) or Hpa‐Tg (cer, *n* = 11) mice; Cerulein + Trehalose treated WT (cer + Treh, *n* = 9) or Hpa‐Tg (cer + Treh, *n* = 19) mice; Cerulein + Trehalose + Aspirin treated WT (cer + Treh + ASP, *n* = 9) or Hpa‐Tg (cer + Treh + ASP, *n* = 16) mice; Cerulein + Trehalose + PG545 treated WT (cer + Treh + PG, *n* = 9) or Hpa‐Tg (cer + Treh + PG, *n* = 18) mice; Cerulein + Trehalose + SST0001 treated WT (cer + Treh + SST, *n* = 9) or Hpa‐ Tg (cer + Treh + SST, *n* = 16) mice. Pancreases samples from the above mentioned groups were collected and weighted. #, *p* < 0.05 ##, *p* < 0.01, ###, *p* < 0.001 compared to cerulein group; $, *p* < 0.05, $$, *p* < 0.01, $$$, *p* < 0.001 compared to combination group.

To reveal the involvement of Hpa in the pathogenesis of AP, we applied a well‐established cerulein‐based mouse model.^12^ Cerulein‐induced pancreatitis was associated with edematous pancreas and significant rises in the serum levels of amylase and lipase in both WT and Hpa‐Tg mice (Figures 1 and 2). The significant elevation in amylase (X3) and lipase (X4) as well as pancreatic edema in response to administration of cerulein to WT mice, were exaggerated in Hpa‐Tg mice indicated by 5 and 7‐fold increase in lipase and amylase serum levels, respectively (Figure 1). Notably, pre‐treatment with PG545 or SST0001, potent Hpa inhibitors, significantly reduced amylase and lipase levels characterizing AP in cerulein‐treated WT and Hpa‐Tg mice and ameliorated pancreatic edema. Similar to PG545 or SST0001, pre‐treatment with Aspirin also reduced pancreatic inflammatory response as demonstrated by decrease in amylase and lipase serum levels in both WT and Hpa‐Tg mice (Figure 1). Noteworthy, combination of Aspirin with either PG545 or SST0001 completely abolished AP at the biochemical level in both subgroups of animals (Figure 1). Likewise, pre‐treatment with Trehalose exerted pancreato‐protective effect against cerulein‐induced AP, evident by reduced amylase and lipase levels and pancreatic edema index (Figure 2). As expected, the combination of Trehalose and Hpa inhibitors was more effective than each drug alone. Combined treatment of Trehalose + PG545 was surpassing the pancreato‐protective effects of Trehalose + SST0001 or Tehalose + Aspirin in terms of lipase and amylase levels in Hpa‐Tg mice. The protective effect of Trehalose alone or in combination with Aspirin or Hpa inhibitors on the pancreatic edema index, reached statistical significance only in Hpa‐Tg mice but not in WT animals. These results indicate that administration of Hpa inhibitors, Aspirin, or Trehalose exerts a protective effect against cerulein‐induced AP, and their combination is more effective than each drug alone.

### 3.2 Histological and ultrastructural analyses

**FIGURE 3:**
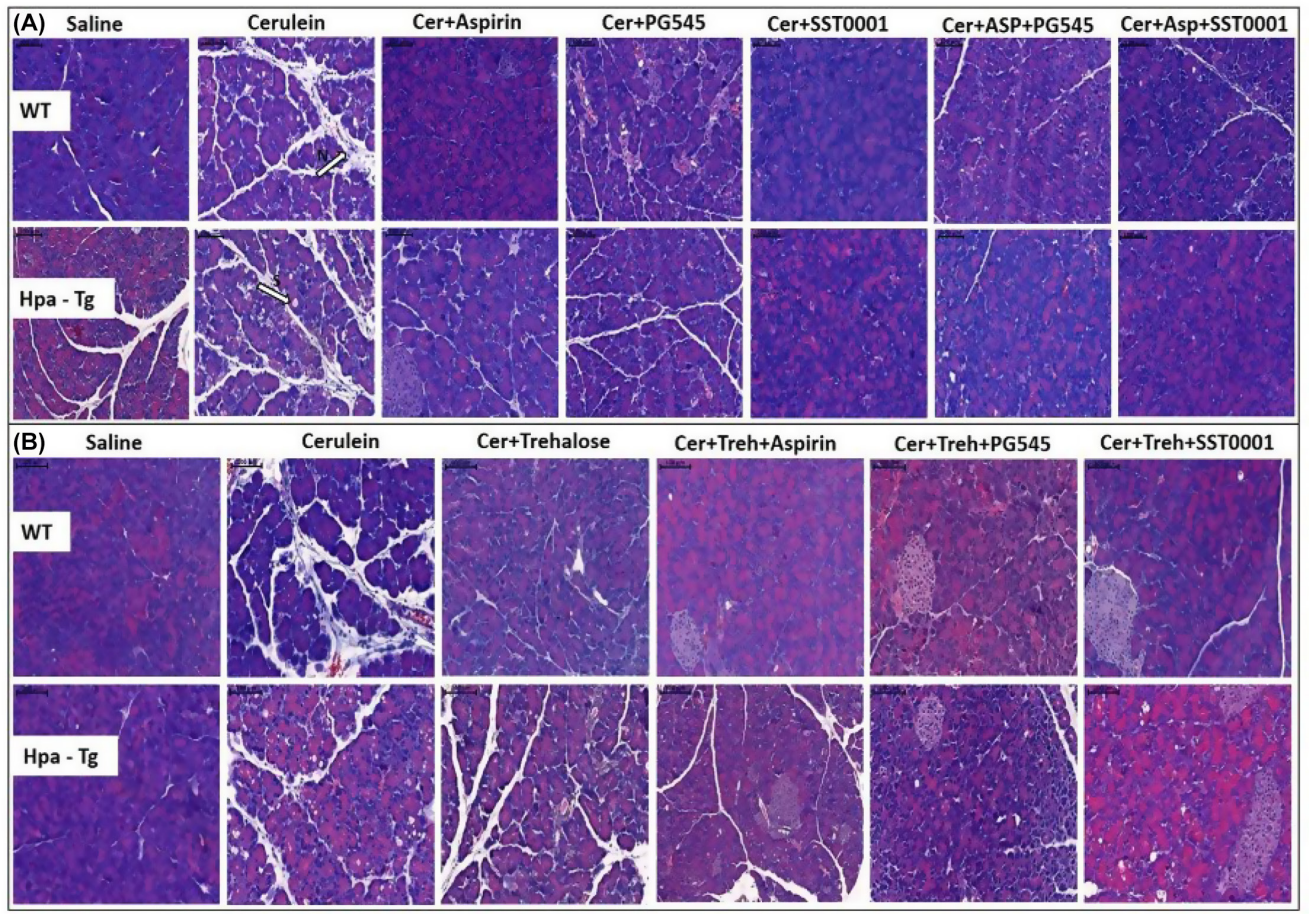
Haematoxylin & Eosin staining. Wild‐type (WT) and heparanase transgenic (Hpa‐Tg) mice were injected with either saline or cerulein in the presence or absence of PG545, SST0001, Aspirin, Trehalose alone, or the indicated combined pre‐treatment. Pancreas tissues were collected 24 h thereafter, and 5‐micron sections of formalin‐fixed, paraffin‐embedded samples were stained with H&E. Shown are representative photomicrographs in scale of 100 μM (A, B). N, infiltration of neutrophils; S, bubble shape bodies.

As hypothesized, injection of cerulein to both WT and Hpa‐Tg mice resulted in morphological changes that characterize AP, such as edema, inflammation and digestive necrosis (Figure 3). Intraperitoneal injection of PG545, SST0001, Aspirin, Trehalose, or combined therapy attenuated the severity of AP as noted by decreased interstitial edema and reduced inflammatory cell infiltration. Specifically, infiltration of neutrophils (appears as purple dots) into the pancreatic tissue was remarkably attenuated by the combined treatment (N, Figure 3A). Histological and cellular damage was observed primarily in the Hpa‐Tg mice treated with cerulein as recognized by white bubble shape bodies (S, Figure 3A).

**FIGURE 4:**
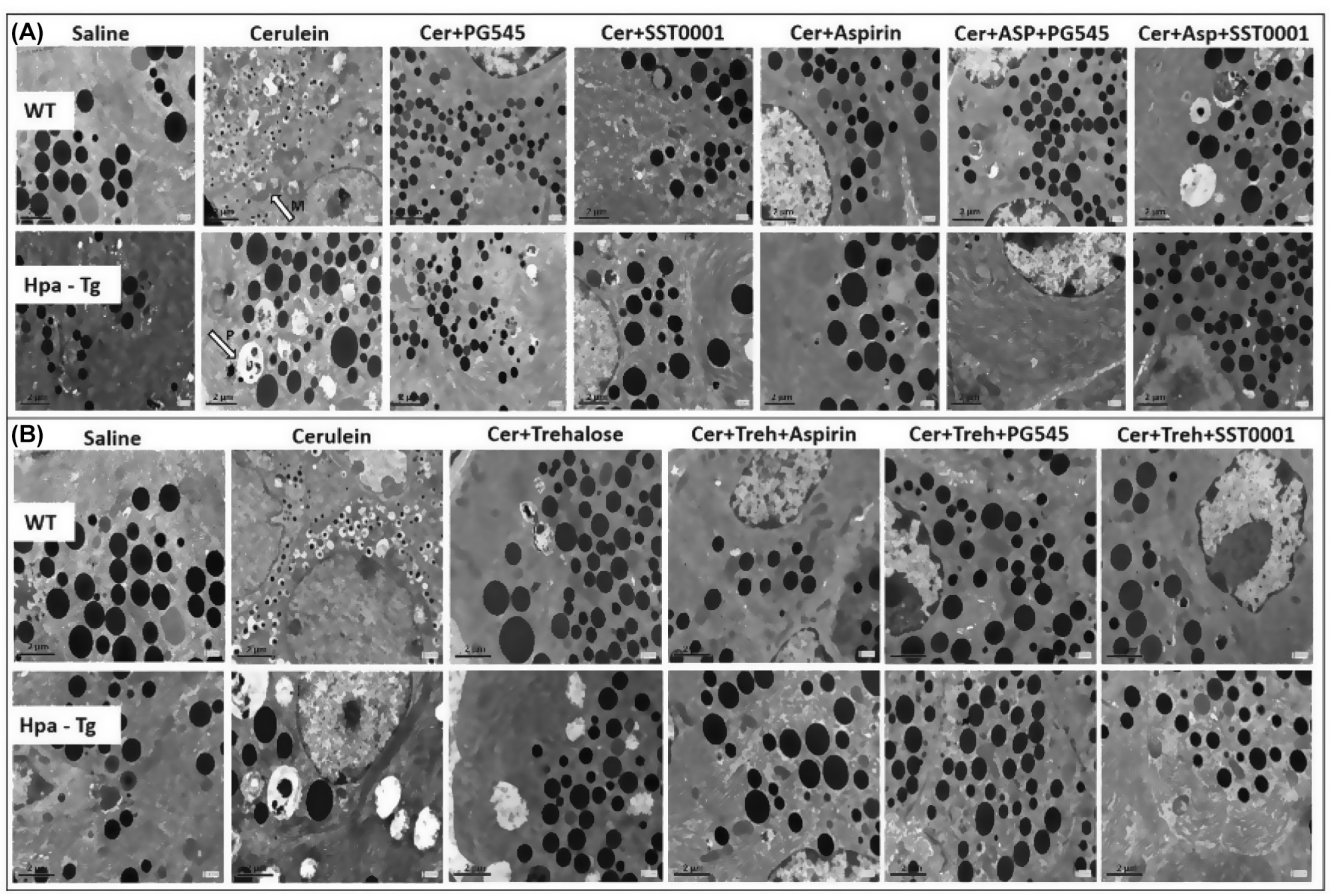
Electron microscopy images. Wild‐type (WT) and heparanase transgenic (Hpa‐Tg mice) were injected with either saline or cerulein in the presence or absence of PG545, SST0001, Aspirin, Trehalose, or the indicated combined pre‐treatment. Pancreas tissues were collected 24 h thereafter and sections were examined with a transmission electron microscope. Shown are representative photomicrographs in scale of 2 μM (A, B). M, mitochondrial vacuolization; P, autophagosome.

Ultrastructural features of pancreatic acinar cells did not show any abnormalities in the control group. Following AP induction in WT mice, the majority of acinar cells demonstrated numerous irregularities (Figure 4). Many mitochondria were edematous with increased translucence of the matrix, and partial destruction of crests (M, Figure 4A). Occasionally, matrix condensation and granulation occurred (Figure 4). Numerous, large autophagosomes containing identifiable cytoplasmic elements, amorphous, membranous or granular masses, and zymogen granules were present within the cytoplasm (P, Figure 4A). Noteworthy, a remarkable increase in zymophagy was noted in Hpa‐Tg mice that were subjected to identical cerulein administration (Figure 4). Intraperitoneal injection of PG545, SST0001, Aspirin or Trehalose attenuated the severity of pancreatitis, and resulted in normalization of the ultrastructural morphology of the acinar cells. Autophagosomes were decreased in number and size. Of notice, E.M images of pancreatic tissue from mice subjected to the combined therapy showed abolishment of the deleterious ultrastructural alterations, revealing a nearly normal ultrastructural appearance, with only a few autophagosomes (Figure 4).

**FIGURE 5:**
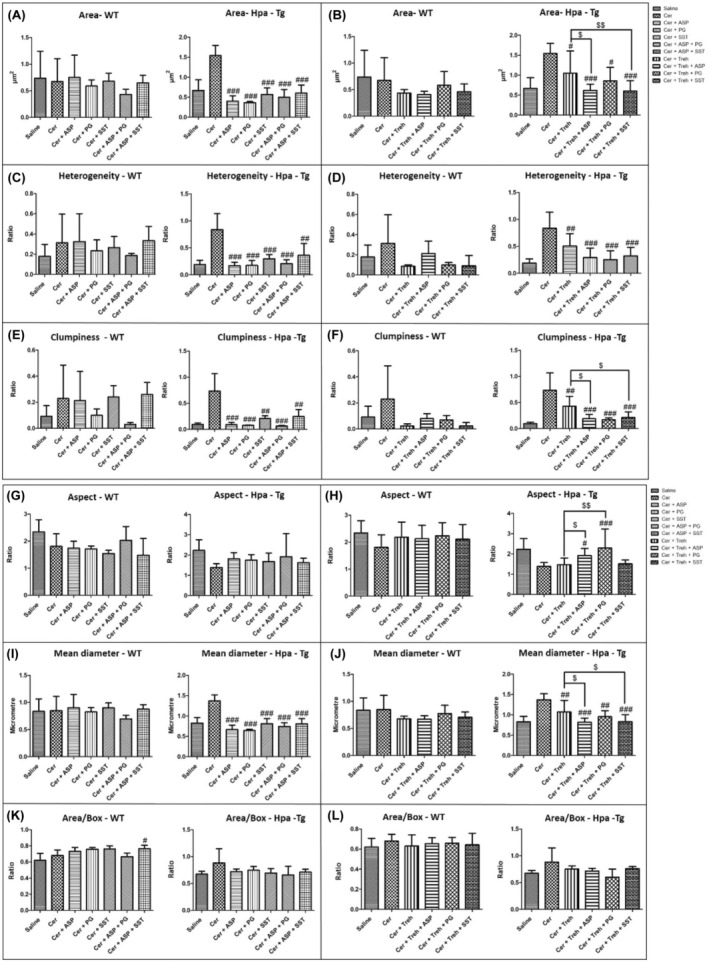
Analysis of electron microscopy images. Wild‐type (WT) and heparanase transgenic (Hpa‐Tg) mice were injected with saline or cerulein in the presence or absence of PG545, SST0001, Aspirin, Trehalose each alone, or the indicated combined pre‐treatment. Pancreas tissues were collected 24 h thereafter. Tissue sections were processed and photographed under a transmission electron microscope and morphometric analysis was performed by Image pro plus 7 software. Mitochondria (*n* = 18–111) were detected and analysed by different features of size, shape, and texture for each sample (A–L) in WT (*n* of fields = 3–10) and Hpa‐Tg mice (*n* of fields = 3–11). Area parameter represents the mitochondria size among the different treatment groups (A, B). Heterogeneity parameter represents the percentage of the pixels that deviate above 10% (C, D). Clumpiness parameter represents clumps accumulation indicating the mitochondrial texture (E, F). Aspect parameter represents the mitochondria shape among the different treatment groups (G, H). Mean diameter parameter represents the mean diameter value of the different mitochondria (I, J). Area/box parameter indicates the mitochondrial shape under normal and stress conditions (K, L). #, *p* < 0.05; ##, *p* < 0.01; ###, *p* < 0.001 compared to cerulein group. $, *p* < 0.05; $$, *p* < 0.01; $$$, *p* < 0.001 compared to combination group.

Morphometric analysis of mitochondria identified quantitative distinctions of size, shape and texture among the treatment groups (Figure 5). Area parameters describing the mitochondria size are presented in Figure 5A,B. Notably, administration of cerulein resulted in profoundly large area size (X3) in Hpa‐Tg mice, suggesting deleterious mitochondrial swelling characterizing AP (Figure 5A,B). Pre‐treatment with PG545, SST0001, Aspirin, Trehalose, or combined therapy significantly reduced area size in WT mice and even more profound in Hpa‐Tg mice (Figure 5A,B). Heterogeneity parameter, which describes the pixel deviation as a result of ultrastructural changes in mitochondria, is depicted in Figure 5C,D. Noteworthy, administration of cerulein resulted in exaggerated heterogeneity (X4) in Hpa‐Tg mice as compared with WT (X1.5) mice, suggesting aberrant mitochondrial changes characterizing AP (Figure 5C,D). While pre‐treatment with PG545, SST0001, Aspirin, and Trehalose (each alone) or combined therapy moderately reduced the heterogeneity parameter in WT mice, it significantly attenuated this parameter in Hpa‐Tg mice (Figure 5C,D). Combined treatment with ASP + SST resulted in aggravated mitochondrial heterogeneity, although this effect did not reach statistical significance as compared to SST0001 or Aspirin alone (Figure 5C,D). Clumpiness parameter, which represents cluster accumulation, was more prominent in Hpa‐Tg (X7) subjected to cerulein‐induced AP as compared with that obtained in WT (X2) mice (Figure 5E,F). Elevated clumpiness level presumably reflects the destruction of mitochondrial crests which results in mitochondrial cluster accumulation (Figure 5E,F). Pre‐treatment with either PG545, SST0001, Aspirin, Trehalose, or combined therapy attenuated the clumpiness parameter compared with c (erulein group in both WT and Hpa‐Tg mice, but reached statistical significance only in Hpa‐Tg animals (Figure 5E,F).

Aspect parameter indicates whether an object tends to be egged shape or round. Low aspect value indicates round mitochondria shape that characterizes mitochondrial swelling (Figure 5G,H). Administration of cerulein resulted in lower aspect value in WT mice and even more significantly lower in Hpa‐Tg mice, suggesting harmful mitochondrial swelling characterizing AP (Figure 5G,H). Pre‐treatment with PG545, SST0001, Aspirin, Trehalose alone or combined therapy elevated the aspect parameter in Hpa‐Tg mice, but to a lesser extent in WT mice (Figure 5G,H). Enhanced aspect parameter was significant only in combined treatment: Trehalose + Aspirin and Trehalose + PG545 when administered into Hpa‐Tg mice (Figure 5H). Mean diameter parameter demonstrates the average diameter value of the different mitochondria in the various treatment groups (Figure 5I,J). Interestingly, administration of cerulein resulted in elevated mean diameter value in WT mice and even more exaggerated (X2) in Hpa‐Tg mice, suggesting aberrant mitochondrial changes characterizing AP (Figure 5I,J). Pre‐treatment with PG545, SST0001, Aspirin, and Trehalose (each alone) or combined therapy reduced the maximal diameter parameter in WT mice, and to a larger extent in Hpa‐Tg mice (*p* value < 0.05) (Figure 5I,J). Area/Box parameter indicates the shape of the mitochondria in the various experimental groups. Low value of area/box indicates irregular shape that usually characterize healthy mitochondria. Increased value of area/box assumingly indicates mitochondrial swelling process due to cellular injury. As expected, cerulein administration resulted in higher area/box value of mitochondria in WT mice and even more prominently in Hpa‐Tg mice (Figure 5K,L). Pre‐treatment with either PG545, SST0001, Aspirin, and Trehalose alone or combined therapy reduced the Area/Box parameter in both WT and Hpa‐Tg mice (Figure 5K,L). Since the severity of the AP was more evident in Hpa‐Tg strain compared to WT, the pancreato‐protective effects of the examined treatment was more remarkable in the E.M analysis of the former strain.

### 3.3 Heparanase activity and expression levels during induction of AP

**FIGURE 6:**
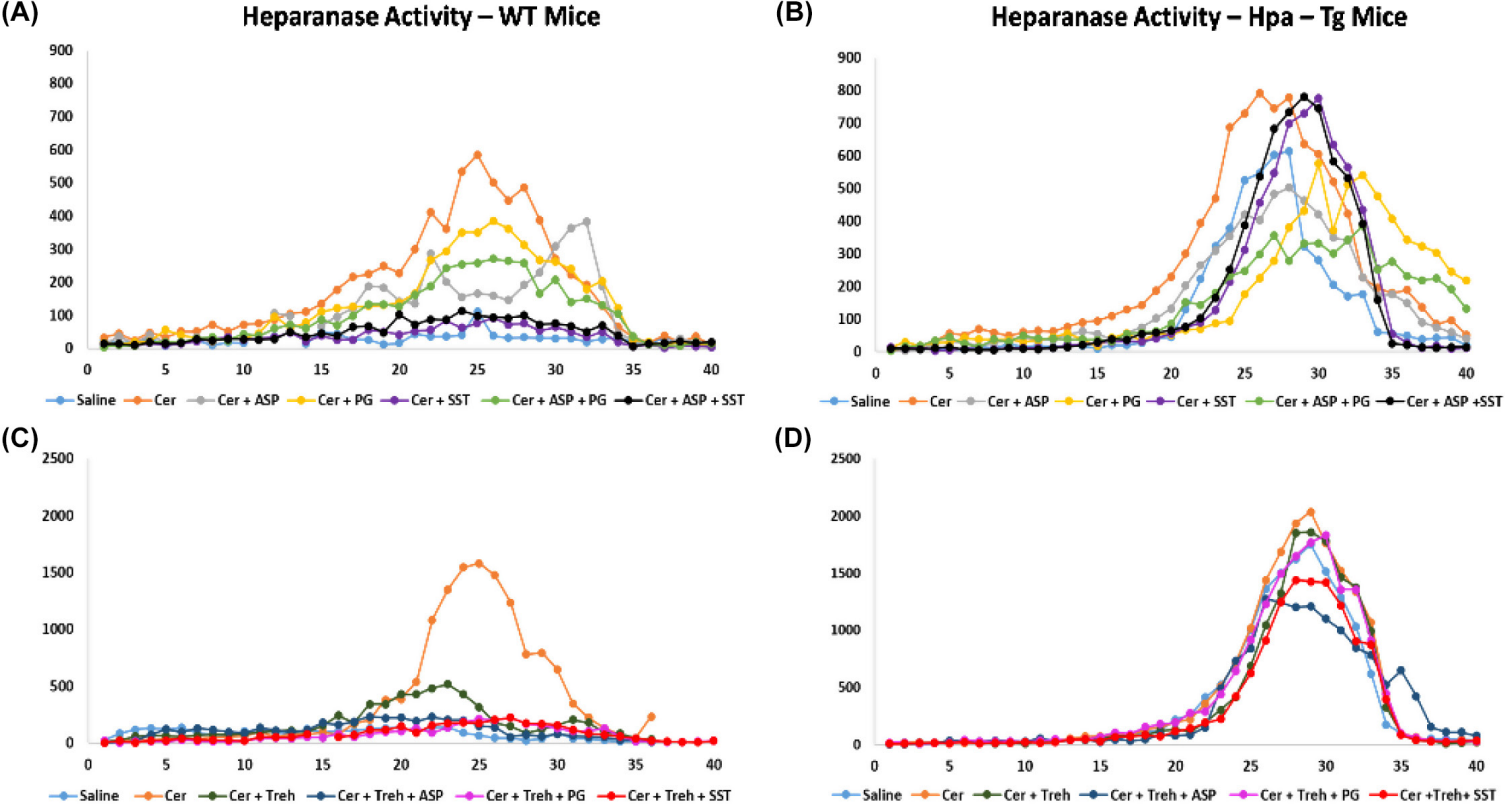
Hpa activity in wild‐type (WT) and heparanase transgenic (Hpa‐Tg) mice. Freshly collected pancreatic tissues were homogenized and incubated on sulphate‐labelled ECM‐coated dishes as described previously.^12^ Hpa activity (release of sulphate‐labelled heparan sulphate degradation fragments) was evaluated in pancreatic tissues harvested from saline, *n* = 3 (light blue); cerulein, *n* = 3 (orange); cerulein + Aspirin, *n* = 3 (grey); cerulein + PG545, *n* = 3 (yellow); cerulein + SST0001, *n* = 3 (purple); cerulein + Aspirin + PG545, *n* = 3 (light green); cerulein + Aspirin + SST0001, *n* = 3 (black); cerulein + Trehalose, *n* = 3 (dark green); cerulein + Trehalose + Aspirin, *n* = 3 (dark blue); cerulein + Trehalose + PG545, *n* = 3 (pink) and cerulein + Trehalose + SST0001, *n* = 3 (red) (A–D).

**FIGURE 7:**
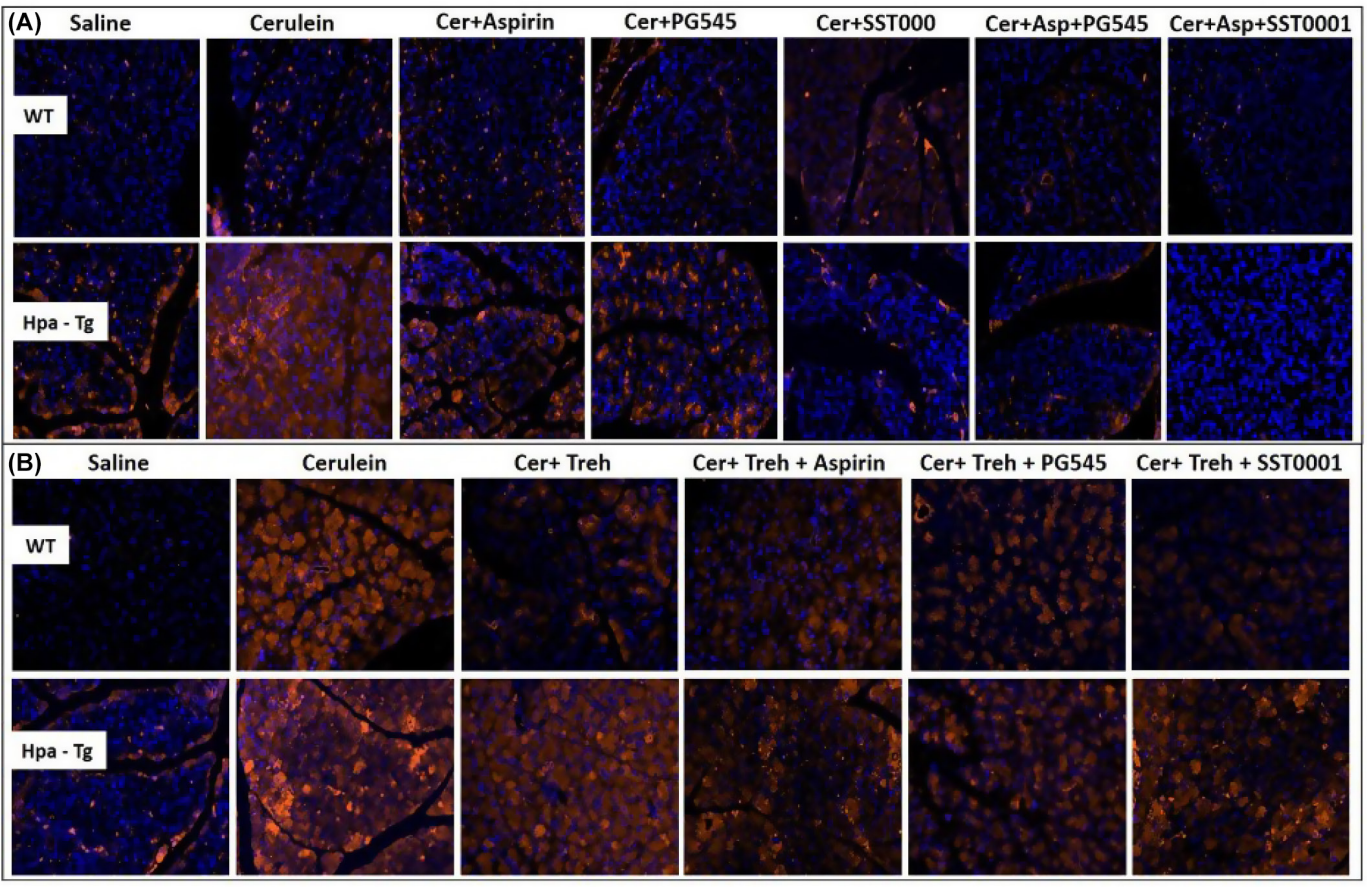
Immunostaining of Hpa. Wild‐type (WT) and heparanase transgenic (Hpa‐Tg) mice were injected with either saline or cerulein in the presence or absence of PG545, SST0001, Aspirin, or Trehalose (each alone) or the indicated combined pre‐treatment. Tissues were harvested, fixed with formalin and embedded in paraffin. 3‐micron sections were subjected to immunostaining with anti‐Hpa antibody (A, B). DAPI staining for DNA appears in blue. All photographs were taken at the same magnification.

**FIGURE 8:**
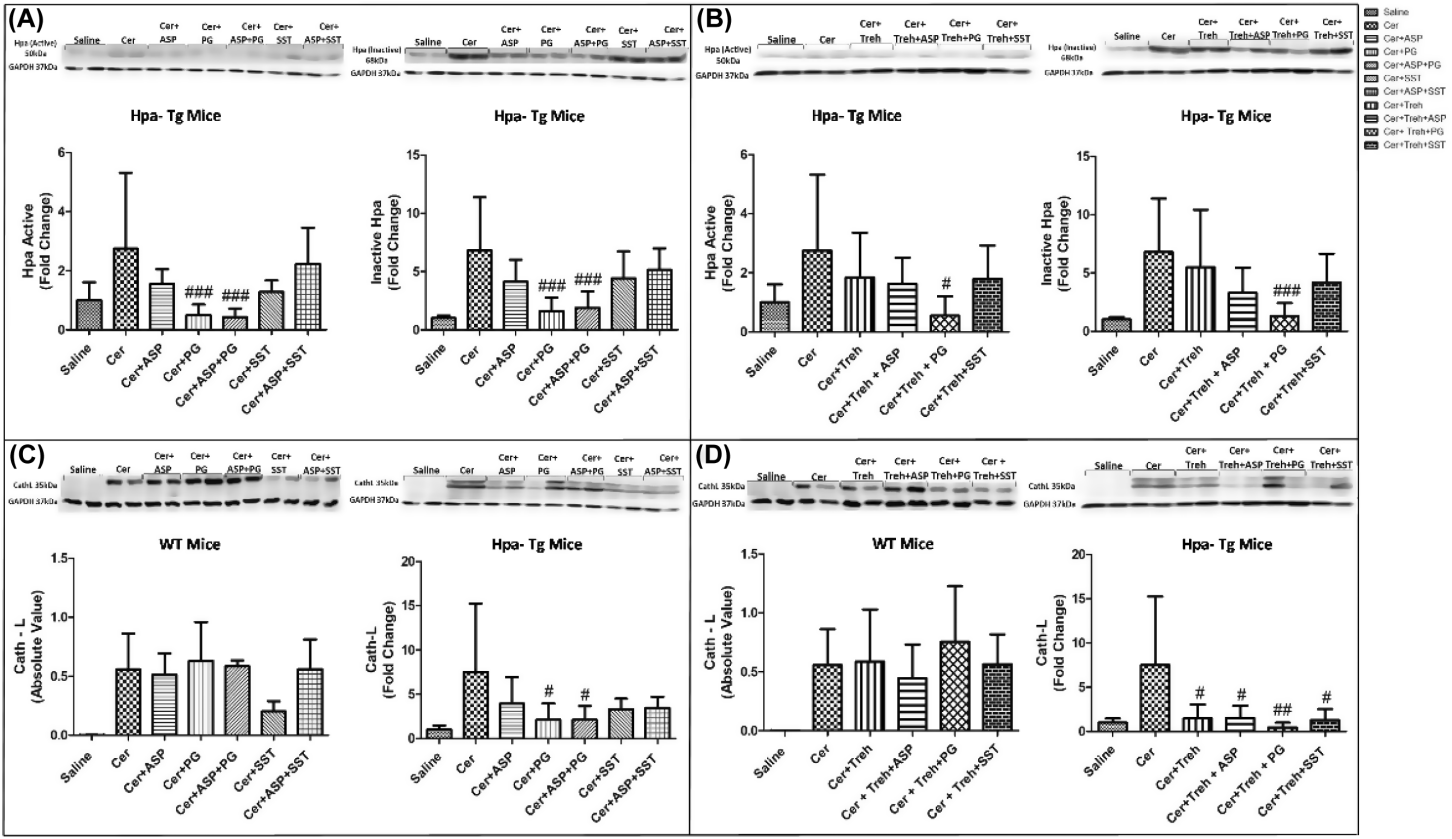
Western blot analysis (Hpa, CathL) of pancreatic tissue extracts derived from wild‐type (WT) and heparanase transgenic (Hpa‐Tg mice). Hpa (A, B) and CathL (C, D) immunoreactive proteins normalized to housekeeping gene GAPDH. Representative Western blots are shown above the densitometry graphs. #, *p* < 0.05; ##, *p* < 0.01; ###, *p* < 0.001 compared to cerulein group. $, *p* < 0.05; $$, *p* < 0.01; $$$, *p* < 0.001 compared to combination group. It should be emphasized that the active form of Hpa (8A), inactive form of Hpa (8A), CathL (8C), p‐STAT3 (10A) and LC3 (10E) in Hpa‐Tg mice were applied on the same representative membrane, therefore were normalized to the same GAPDH. Moreover, also in Trehalose experimental groups, the active form of Hpa (8B), inactive form of Hpa (8B), CathL (8D), p‐STAT3 (10B) and LC3 (10F) in Hpa‐Tg mice were applied on the same representative membrane, therefore were normalized to the same GAPDH. In the WT mice, CathL (8D) and p‐STAT3 (10B) were applied on the same representative membrane, therefore were normalized to the same GAPDH.

In order to examine further the involvement of Hpa in the pathogenesis of AP, we performed Hpa activity assay, anti‐Hpa immunofluorescent staining and Western blot analysis of Hpa protein in the pancreatic tissue of WT and Hpa‐Tg mice. Notably, Hpa enzymatic activity was significantly elevated X6 (Figure 6A) and X15 (Figure 6C) in cerulein‐treated WT mice. The high Hpa activity observed following cerulein treatment was abrogated by Aspirin, PG545, SST0001 and Trehalose in WT mice and even more so by combining either Aspirin and PG545, Aspirin and SST0001, Trehalose and Aspirin, Trehalose and PG545 or Trehalose and SST0001 (Figure 6A,C). As expected, Hpa activity in untreated Hpa‐Tg pancreas was much higher than in WT pancreas and was significantly (X1.5) elevated following cerulein administration (Figure 6B,D). A marked reduction was noted in response to Aspirin and PG545, but there was no effect to SST0001 or Trehalose alone. Combination treatment with Aspirin and PG545, Trehalose and Aspirin, Trehalose and PG545, or Trehalose and SST0001 reduced Hpa activity more effectively than each drug alone, whereas the combination of Aspirin and SST0001 was not effective (Figure 6B,D). The latter finding could be attributed to potential competition of Aspirin and SST0001 on Hpa, especially when the enzyme is overexpressed, thus reducing their efficacy of as compared to each drug alone. Immunofluorescent staining revealed a significant increase in Hpa immunoreactivity in the pancreatic tissue of WT and Hpa‐Tg mice that were subjected to AP (Figure 7). As demonstrated, AP is characterized by Hpa elevation in both WT and Hpa‐Tg mice, yet its upregulation was more remarkable in the latter (Figure 7A,B). Notably, pre‐treatment with either Aspirin, PG545, SST0001 or Trehalose reduced the upregulation of Hpa (Figure 7A,B). Noteworthy, combination of either Aspirin and PG545, Aspirin and SST0001, Trehalose and Aspirin, Trehalose and PG545 or Trehalose and SST0001 reduced Hpa upregulation (Figure 7A,B). In line with the above Hpa activity and immunostaining results, Western blot analysis revealed that pancreatic Hpa expression was significantly elevated in Hpa‐Tg mice following cerulein‐induced AP (Figure 8A). Hpa protein expression was reduced by Aspirin, PG545, SST0001 or Trehalose when given alone and affectedly declined when these agents were administered in combination (Figure 8A). Densitometric analysis of the immunoblotting results revealed a significant increase in the active (50 kDa) and latent inactive (65 kDa) forms of Hpa proteins in pancreata of cerulein‐treated Hpa‐Tg mice (X2.5 and X7 respectively) (Figure 8A,B). Notably, this elevation was markedly reduced by Aspirin or Trehalose and to a larger extent by the Hpa inhibitors PG545 and SST0001, and their combination with Aspirin or Trehalose in Hpa‐Tg mice (Figure 8A,B). Latent Hpa is converted to its active 50 + 8 kDa form upon the removal of a linker segment by CathL.^24^ Similar to Hpa, immunoreactivity of CathL was enhanced in both WT and Hpa‐Tg mice treated with cerulein (Figure 8C,D).

### 3.4 Cytokines expression during induction of AP

**FIGURE 9:**
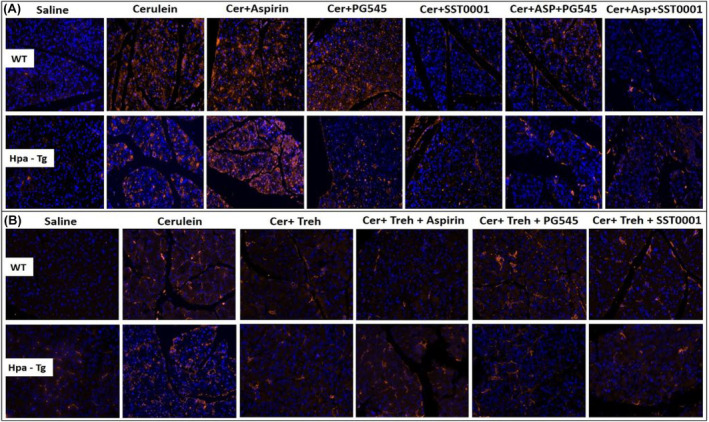
Immunostaining of F4/80. Wild‐type (WT) and heparanase transgenic (Hpa‐Tg) mice were injected with either saline or cerulein in the presence or absence of PG545, SST0001, Aspirin, Trehalose alone, or the indicated combined pre‐treatment. Tissues were harvested, fixed with formalin and embedded in paraffin. 3‐micron sections were subjected to immunostaining with anti‐F4/80 – a macrophage‐specific marker (A, B). DAPI staining for DNA appears in blue. All photographs were made at the same magnification.

**FIGURE 10:**
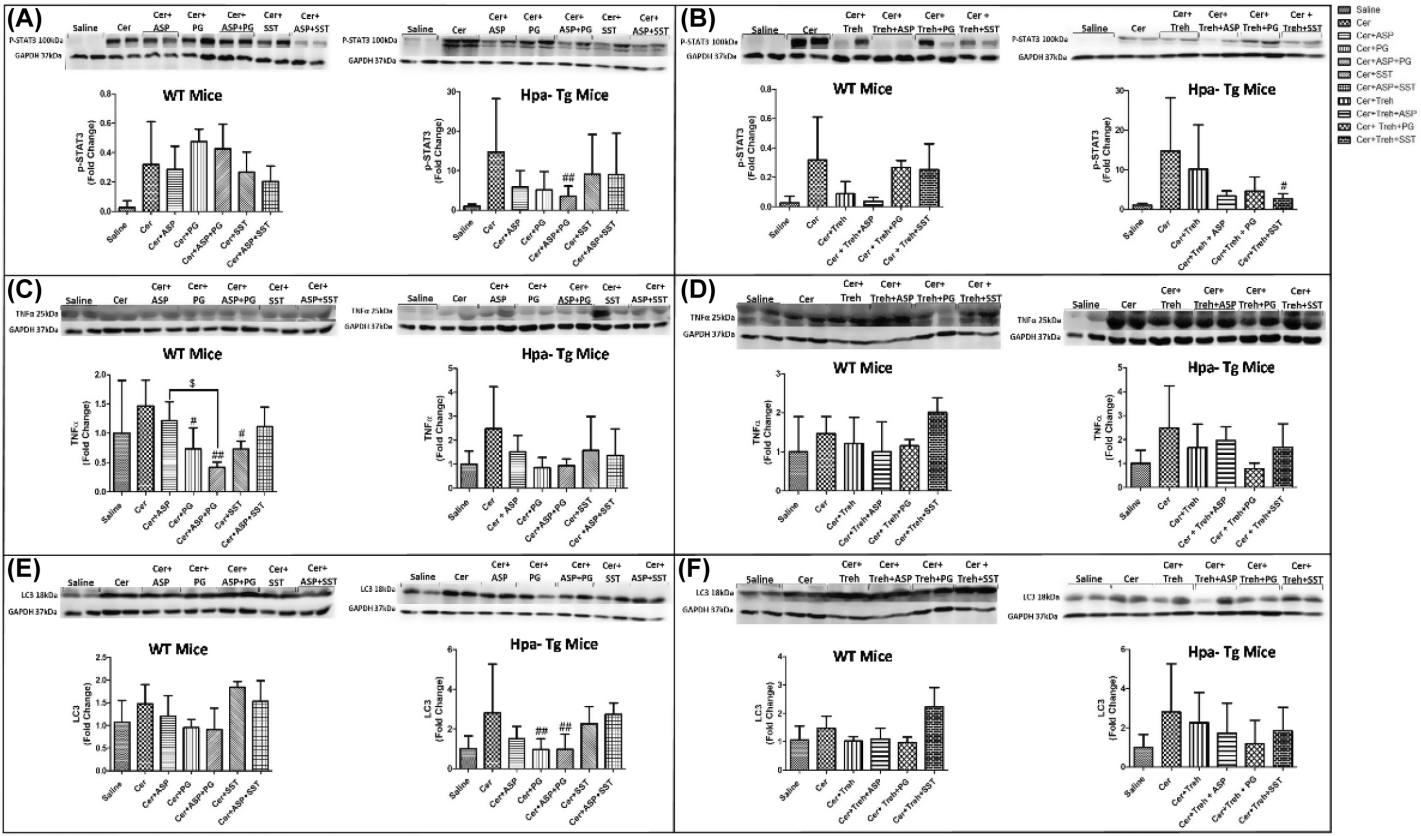
Western blot analysis (biomarkers of inflammation) of pancreatic tissue extracts derived from wild‐type (WT) and heparanase transgenic (Hpa‐Tg mice). p‐STAT3 (A, B), TNF‐α (C, D) and LC3 (E, F) immunoreactive proteins normalized to housekeeping gene GAPDH. Representative Western blots are shown above the densitometric graphs. Cerulein induction leads to the activation of inflammatory cascade with elevated abundance of p‐STAT3, TNF‐α, and LC3 in WT (*n* = 3–6) and Hpa‐Tg (*n* = 4–16) mice. #, *p* < 0.05; ##, *p* < 0.01; ###, *p* < 0.001 compared to cerulein group; $, *p* < 0.05, $$, *p* < 0.01, $$$, *p* < 0.001 compared to combination group. It should be emphasized that TNF‐α (10C) and LC3 (10E) in WT mice were applied on the same representative membrane, therefore were normalized to the same GAPDH. Moreover, also in Trehalose experimental groups, TNF‐α (10D) and LC3 (10F) in WT mice were applied on the same representative membrane, therefore were normalized to the same GAPDH.

Immunofluorescent staining revealed a significant increase in macrophage infiltration into the pancreatic tissue of WT and Hpa‐Tg mice that were subjected to AP, though the increase was much more profound in the Hpa‐Tg mice (Figure 9). Of note, the pattern of macrophage staining strongly resembled that of neutrophils infiltration (Figure 9), suggesting an interplay between Hpa, macrophage abundance and TNF‐α production during AP. Support for this notion was derived from our findings that pre‐treatment with Aspirin, PG545, SST0001, Trehalose, or their combination, markedly reduced the upregulation of Hpa (Figure 7) along macrophage infiltration in most experimental groups. Monotherapy with PG545 or Aspirin did not reduce macrophage abundance compared with combined therapy (Figure 9A,B). In line with these changes, the levels of p‐STAT3 (Figure 10A,B), TNF‐α (Figure 10C,D), and LC3 (Figure 10E,F) which reflect the inflammatory status and extent of autophagy, respectively, were also augmented in the pancreatic tissue of cerulein‐treated WT and Hpa‐Tg mice. Noteworthy, pre‐administration of Aspirin alone or combined with Hpa inhibitors, PG545 and SST0001, resulted in an inhibitory effect on the immunoreactive levels of the above mentioned key proteins, although did not always reached statistical significance. Specifically, the abundance of Hpa (Figure 8A,B), CathL (Figure 8C,D), p‐STAT3 (Figure 10A,B), TNF‐α (Figure 10C,D) and LC3 (Figure 10E,F) was elevated during AP and slightly reduced following Trehalose treatment either alone or combined with Aspirin or Hpa inhibitors in both the WT and Hpa‐Tg. Surprisingly, combined treatment with Trehalose+SST0001 in WT mice enhanced immunoreactive levels of TNF‐α and LC3 (Figure 10D,F), although this effect did not reach statistical significance as compared to SST0001 or Aspirin alone (Figure 10D,F). Thus, Hpa induction by cerulein is associated with activation of key signalling pathways involved in the promotion of AP, whereas Aspirin, Trehalose and Hpa inhibitors efficiently abrogate the damage, especially when combined.

### 3.5 mRNA expression of inflammatory key mediators associated with AP

**FIGURE 11:**
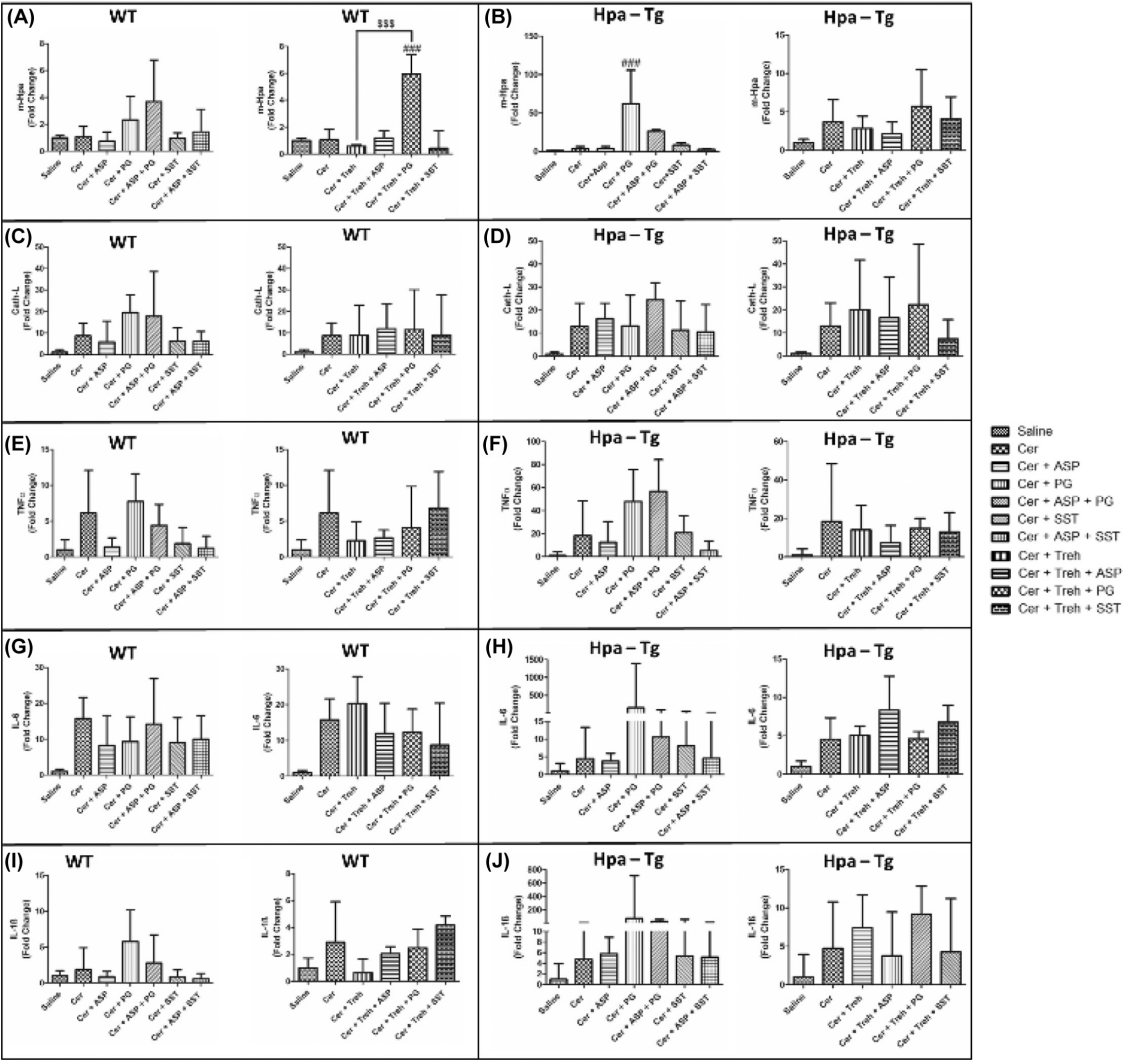
Expression of inflammatory cytokines during acute pancreatitis (AP) in wild‐type (WT) and heparanase transgenic (Hpa‐Tg mice). Total RNA was extracted from WT (*n* = 3–10) and Hpa‐Tg mice (*n* = 3–11) corresponding pancreas tissues and subjected to real‐time qPCR analyses applying primers specific for Hpa (A, B), CathL (C, D), TNFα (E, F), IL‐6 (G, H) and IL‐1β (I, J). Relative gene expression (fold‐change) is shown graphically in relation to the levels in control pancreas as set arbitrarily to a value of 1. #, *p* < 0.05; ##, *p* < 0.01; ###, *p* < 0.001 compared to cerulein group. $, *p* < 0.05; $$, *p* < 0.01; $$$, *p* < 0.001 compared to combination group.

Total RNA extracted from the pancreas of untreated and treated WT and Hpa‐Tg mice was subjected to quantitative real‐time PCR applying the relevant primers. Hpa expression remained the same in WT mice and was elevated X4 in cerulein‐treated Hpa‐Tg mice compared to the saline group (Figure 11A,B). Noteworthy, pre‐treatment with Aspirin or SST0001 alone reduced Hpa levels compared with untreated AP‐mice. Unexpectedly, monotherapy with PG545 or PG545 with Aspirin caused over expression of Hpa‐mRNA levels in both WT and Hpa‐Tg mice (Figure 11A,B). This behaviour may represent a compensatory response to the decline in the immunoreactive levels. In line with these changes, the expression of CathL was increased in the pancreatic tissue of WT mice (X9) and even more so (X13) in Hpa‐Tg mice following cerulein administration as compared to the saline subgroup (Figure 11C). Pre‐treatment with Aspirin, SST0001 or Aspirin + SST0001 reduced CathL mRNA levels compared with cerulein‐treated mice, whereas PG545 or PG545 + Aspirin elevated CathL levels in both WT and Hpa‐Tg mice presumably due to activation of compensatory feedback mechanism (Figure 11C). Likewise, pre‐administration of Trehalose alone or combined with either Aspirin, PG545 or SST0001 resulted in the comparable effect in both subgroups of mice (Figure 11). AP is characterized by the secretion of various key cytokines including TNFα, IL‐6, and IL1‐β. These inflammatory mediators were therefore measured following treatment with either Aspirin, PG545, SST0001, Trehalose or the combined therapy. In line with Hpa and CathL alternations, the various treatments resulted in a similar trend in the expression levels of the studied cytokines (Figure 11E,G,I). Specifically, PG545 monotherapy or combined with Aspirin enhanced the expression of TNFα, IL‐6, and IL1‐β in WT and Hpa‐Tg mice. A similar effect was exerted by pre‐administration of Trehalose alone or combined with either Aspirin, PG545 or SST0001 (Figure 11F,H,J).

### 3.6 Effects of aspirlose on the severity of AP

**FIGURE 12:**
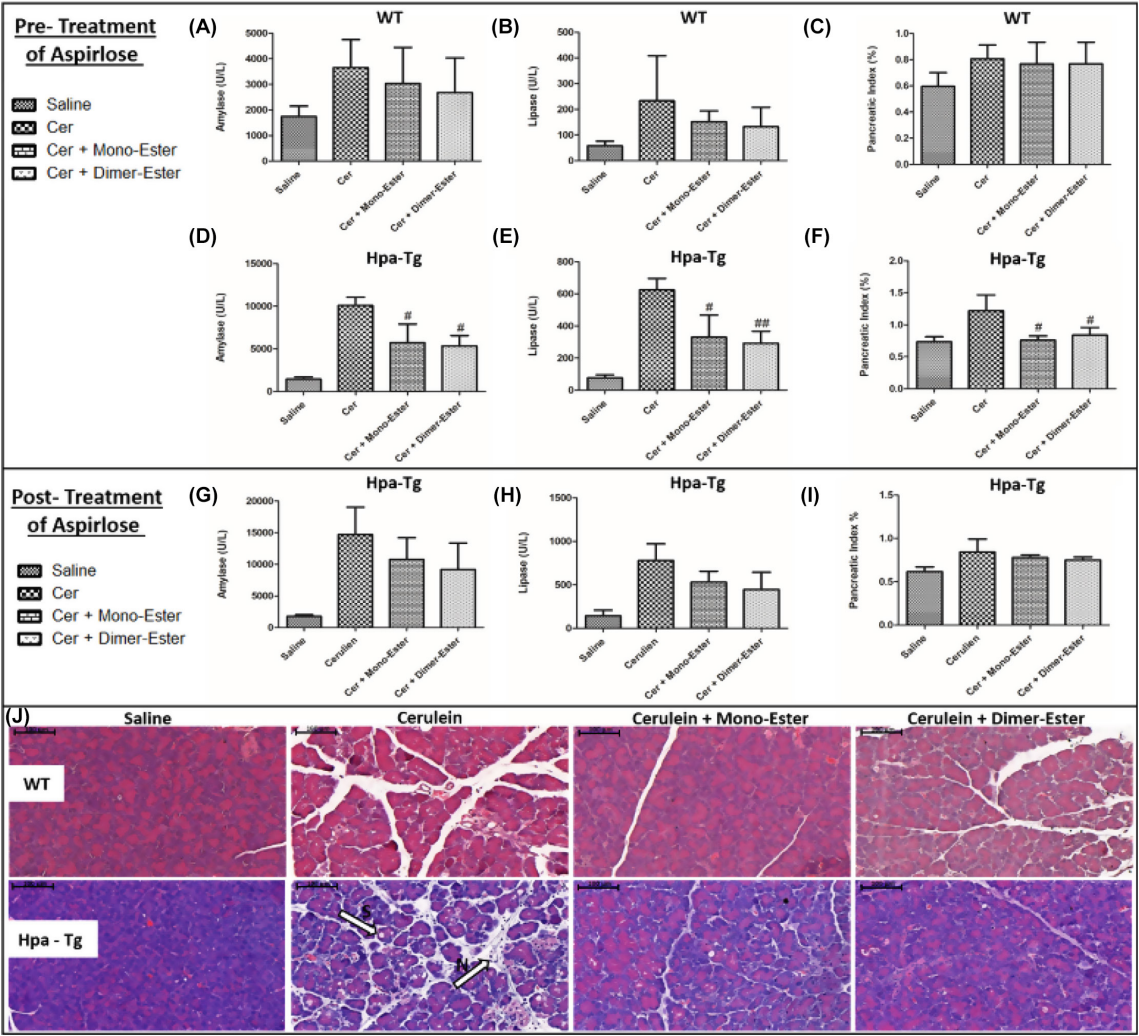
Effects of Aspirlose on experimental AP in wild‐type (WT) and heparanase transgenic (Hpa‐Tg) mice. Upper panel describes serum levels of amylase (A, D), lipase (B, E) and pancreatic index (pancreas/body weight ratio) (C, F) in WT and Hpa‐Tg mice subjected to cerulein‐induced AP. Blood samples were collected and evaluated biochemically for lipase and amylase levels from control untreated WT (saline, *n* = 6–7) or Hpa‐Tg (saline, *n* = 3) mice; cerulein treated WT (cer, *n* = 8–9) or Hpa‐Tg (cer, *n* = 3) mice; Cerulein + Aspirlose Mono‐ester treated WT (cer + Mono‐ester, *n* = 8) or Hpa‐Tg (cer + Mono‐ester, *n* = 3) mice; Cerulein + Aspirlose Dimer‐ester treated WT (cer + Dimer‐ester, *n* = 8) or Hpa‐Tg (cer + Dimer‐ester, *n* = 3) mice. Pancreases samples from the above mentioned groups were collected and weighted. #, *p* < 0.05; ##, *p* < 0.01; ###, *p* < 0.001 compared to cerulein group. $, *p* < 0.05; $$, *p* < 0.01; $$$, *p* < 0.001 compared to combination group. Middle panel describes serum levels of amylase (G), lipase (H) and pancreatic index (pancreas/body weight ratio) (I) in Hpa‐Tg mice subjected to cerulein‐induced AP. Blood samples were collected and evaluated biochemically for lipase and amylase levels from control untreated Hpa‐Tg (saline, *n* = 4) mice; cerulein treated Hpa‐Tg (cer, *n* = 4) mice; Cerulein + Aspirlose Mono‐ester treated Hpa‐Tg (cer + Mono‐ester, *n* = 4) mice; Cerulein + Aspirlose Dimer‐ester Hpa‐Tg (cer + Dimer‐ester, *n* = 4) mice. Pancreases samples from the above mentioned groups were collected and weighted. #, *p* < 0.05; ##, *p* < 0.01; ###, *p* < 0.001 compared to cerulein group. $, *p* < 0.05; $$, *p* < 0.01; $$$, *p* < 0.001 compared to combination group. Lower panel presents H&E staining of WT and Hpa‐Tg mice injected with either saline or cerulein in the presence or absence of Aspirlose as mono‐ester or dimer‐ester (J). N, infiltration of neutrophils; S, bubble shape bodies. Shown are representative photomicrographs in scale of 100 μM (J).

In light of the pancreatic‐protective effects of Aspirin and Trehalose alone, it is appealing to synthesize a novel compound which combines both Aspirin and Trehalose and may possess pharmacological and therapeutic properties over each component alone. As demonstrated earlier, cerulein‐induced AP in WT (*n* = 6–9) mice was associated with significant rises in the serum levels of amylase (X2) and lipase (X3) (Figure 12A,B). These increases were associated with enhancement of pancreatic edema index and tissue inflammation response (Figure 12C). Moreover, histological staining revealed in cerulein‐treated mice infiltration of neutrophils (appears as purple dots) into the pancreatic tissue (N, Figure 12J) and cellular punctures observed mainly in the Hpa‐Tg mice as recognized by white bubble shape bodies (S, Figure 12J). All types of responses to administration of cerulein were more profound in Hpa‐Tg mice (*n* = 3–4), as evident by 6 and 5‐fold increases in lipase and amylase levels, respectively (Figure 12D,E) and augmented pancreatic edema index (Figure 12F). Aspirlose administration as pre‐treatment exerted comparable pancreato‐protective effect in both subgroups of mice, yet this encouraging result on amylase, lipase, and pancreatic index were more remarkable and significant in Hpa‐Tg mice (Figure 12D,E,F) as compared to WT animals (Figure 12A,B,C). In light of the protective effects of Aspirlose as pre‐treatment, we examined also post‐treatment administration. Specifically, post‐treatment with Aspirlose in both forms was conducted against ceruline‐induced AP in Hpa‐Tg mice (Figure 12G,H,I). Administration of cerulein into Hpa‐Tg mice (*n* = 4), increased lipase and amylase levels by 5 and 7.5‐fold, respectively (Figure 12G,H) as well as elevated pancreatic edema index (Figure 12I). Notably, post‐treatment of Hpa‐Tg mice with Aspirlose (in both forms) attenuated amylase and lipase levels and pancreatic edema index (Figure 12G,H,I). Collectively, due to the protective action of both Aspirin and Trehalose against AP, we synthesized a new compound that combines both ingredient agents, termed Aspirlose, which was pancreato‐protective when given as pre and post‐treatment (Figure 12).

## 4 DISCUSSION

Acute pancreatitis is characterized by significant morbidity and mortality. While mild AP is without serious complications, mortality from pancreatitis is approximately 1% overall^26^, ^27^ and may reach among hospitalized patients as high as 30%–40%.^28^ Despite intensive investigation, neither the etiology nor the pathophysiology of the pancreatitis process is fully understood and there is no specific or effective pharmacological treatment to prevent clinical progression of the disease or death.^29^, ^30^ Here, we extend our previous findings that Hpa is engaged in AP,^12^ as evident by upregulation of its expression and activity in cerulean‐induced AP. Furthermore, treatment with Hpa inhibitors, namely Pixatimod (PG545) or Roneparstat (SST0001), significantly attenuated lipase and amylase elevation, pancreatic edema, recruitment of neutrophils, induction of cytokines (i.e. TNFα, IL‐6), and activation of STAT3 signalling. The pancreato‐protective effects of PG545 and SST0001 strongly implicate Hpa in the pathogenesis of AP. This notion is further supported by our observation that all the above adverse characteristics of AP were even more prominent in Hpa‐Tg mice endowed with the higher levels of Hpa in their pancreas, suggesting that the severity of AP correlates with Hpa levels. Furthermore, in agreement with our previous findings^12^ we found that cerulein‐induced AP is associated with upregulation of CathL which likely contributes to the disease. The adverse involvement of CathL in the pathogenesis of AP is supported by reports demonstrating that the severity of pancreatitis is reduced in CathL‐knock out (KO) mice,^31^ or when it was inhibited by pharmacological means.^32^ In this context, CathL functions not only to damage the pancreas by its digestive activity but also by its ability to activate latent Hpa.^12^ The latter is first synthesized as a readily secreted inactive 65 kDa pro‐enzyme that is taken up by the cells and transferred to late endosomes/lysosomes.^33^, ^34^ In the lysosomes, Hpa is proteolytically processed by CathL into its active form, a heterodimer constituted of 8 kDa and 50 kDa subunits.^35^, ^36^, ^37^ In addition, latent Hpa can get activated extracellularly by secreted CathL. Thus, the induction of Hpa expression in the course of AP is accompanied by enhanced expression of CathL which, in turn, activates Hpa in a loop that feeds itself, generating continuous production of active Hpa that functions to support AP. This devastating loop is efficiently blocked, nonetheless, by the Hpa inhibitors Pixatimod and Roneparstat, lending hope that these compounds, now in phase I/II clinical trials in cancer patients^38^, ^39^ will prove efficacious also in AP.

In the current study, we also demonstrated that both Aspirin and Trehalose alone or in combination with the above Hpa inhibitors effectively mitigate AP more than each drug alone, thus may constitute a novel therapy for this common orphan disease. Notably, the protective effects of either Treahlose, Aspirin, PG545, SST0001 alone or in combination, were more prominent mainly in Hpa‐Tg mice as compared with WT animals. This behaviour could be attributed to the fact that Hpa‐Tg mice were more sensitive to AP and the manifestations of the disease were more severe in this strain. The pancreato‐protective effect of Aspirin, the most prominent representative of NSAIDs, is not surprising. PGs production is mediated by the activity of COXs, which exist as distinct two isoforms referred to as COX‐1 and COX‐2.^9^ While COX‐1 is constitutively expressed in most tissues and is the major source of housekeeping PGs, COX‐2, key enzyme responsible for the generation of PGs, leukotrienes, and thromboxane, is induced by pro‐inflammatory stimuli.^9^, ^40^ NSAIDs are inhibitors of both COX1 and 2, thus avert PGs production and could reduce the inflammatory response characterizing AP both clinically and experimentally.^41^ In this respect, nonselective NSAIDs are used to prevent ERCP pancreatitis, given their efficacy, safety, availability and affordability,^42^, ^43^ although their efficacy in reducing the incidence and severity of post‐ERCP pancreatitis (PEP) was not confirmed when given as a pre‐treatment.^44^ In humans, the efficacy of nonselective NSAIDs in preventing the clinical progression of AP was not established thoroughly. Specifically, while clinical studies evaluated the analgesic effect and safety profile of nonselective NSAIDs on subjects with AP,^45^, ^46^ no controlled studies evaluated the efficacy of nonselective NSAIDs in preventing the clinical progression of AP. In this respect, a recent study examined the impact of Indomethacin therapy in subjects with AP who had systemic inflammatory response syndrome (SIRS) at the time of enrollment.^47^ While rectal indomethacin can be safely administered over 48 h, it is not superior to placebo in reducing the SIRS or clinical progression in a high‐risk population with AP.^47^ In contrast to the inconsistent findings of the clinical trials, experimental studies applying animal models showed that the administration of indomethacin or diclofenac after AP induction decreased disease severity and mortality.^48^, ^49^, ^50^, ^51^, ^52^ Our results clearly show that pre‐treatment with Aspirin significantly attenuated the severity of AP in both WT and Hpa‐Tg mice as was evident by reduction in pancreatic enzymes and edema along histological improvement. It is well known that the onset of AP is associated with an exaggerated immune response that plays a critical role in the pathogenesis of severe AP and may represent a potential therapeutic target for AP. The initial parenchymal injury activates inflammatory cells and transcription factors, which lead to the production of various pro‐inflammatory cytokines, such as TNF‐α, IL‐1, IL‐6, and IL‐8,^7^, ^8^ as observed in the present study. The pro‐inflammatory milieu induces COX‐2, a key enzyme in the conversion of arachidonic acid to PGs, leukotrienes, and thromboxanes.^9^ These inflammatory mediators are released into the circulation, where they contribute to the development of SIRS and multi‐organ failure, and even death.^53^, ^54^ The pancreato‐protective effects of Aspirin could be attributed to its anti‐inflammatory properties as was evident by reducing macrophage infiltration into the pancreatic tissue, and the downregulation of cytokines along with Hpa. However, the finding that Aspirin possesses a partial inhibitory effect against Hpa,^16^ suggests that this new mode of action may contribute to the protective effects of Aspirin against AP. However, our findings that Aspirin, even when administered at high doses, did not affect Hpa activity in both WT and Hpa‐Tg mice, argue against this possibility and hint that its pancreato‐protective effect is mainly due its anti‐inflammatory action. Yet, our findings that the effect of certain treatments, especially PG545, on the expression of key cytokines are not always in line with their inhibitory action on the immunoreactive levels of these cytokines. Specifically, in some cases the expression of certain cytokines was enhanced while their immunoreactive proteins declined (Figures 10 and 11). These conflicting findings could be explained by negative feedback phenomenon, where RNA expression and immunoreactivity are not always overlapping and sometime even opposing. Additionally, this discrepancy could be due to the different actions of the tested drugs, where some of them possess Hpa inhibitory actions, others are known to exert anti‐inflammatory or anti‐oxidative affects. Therefore, combination of the tested drugs yielded mixed results reliant on their prominent action. In this context, PG545 shows various action such as inhibition of growth factor release, yet it may stimulate the innate immune response,^38^ thus augmenting the expression of some cytokines.

The current study shows that pre‐treatment with Trehalose alone or in combination with Aspirin, PG545, or SST0001 successfully ameliorate AP in both WT and Hpa‐Tg mice. These results are in agreement with a previous report by Biczo et al^19^ demonstrating that Trehalose exerted pancreato‐protective action in cerulein‐induced AP mouse model. These beneficial effects of Trehalose were evident by abrogating mitochondrial dysfunction, endoplasmic reticulum (ER) stress, and impaired autophagy. Furthermore, administration of Trehalose largely prevented trypsinogen activation, necrosis, and other parameters of pancreatic injury due to cerulein or other models of AP. In line with these results, we report that administration of cerulein resulted in profound increase mitochondrial area in Hpa‐Tg mice. Noteworthy, administration of cerulein also resulted in exaggerated mitochondrial heterogeneity and clumpiness in Hpa‐Tg as compared with WT mice. Pre‐treatment with Trehalose as well as PG545, SST0001, Aspirin, or combined therapy reduced these adverse mitochondrial alterations in WT mice and even more profoundly in Hpa‐Tg mice. Since the severity of the AP is more severe in Hpa‐Tg strain compared to WT, the pancreato‐protective effects of the examined treatment were more evident in Hpa‐Tg mice as was evident by the E.M analysis results. Interestingly, combined treatment with Aspirin+SST0001 (ASP+SST) resulted in enhanced mitochondrial heterogeneity, yet this stimulatory effect was not statistically significant as compared with the effect of each drug alone. We assume that the observed stimulatory action of Aspirin+SST0001 may stem from high variability as was evident by standard deviation rather than real phenomenon. Taken together, these findings suggest a critical role for mitochondrial dysfunction in the pathogenesis of AP. Support for this concept was derived from Biczo at al^19^ who demonstrated that acinar cell mitochondrial dysfunction is implicated in the pathogenesis of pancreatitis and that restoration of mitochondrial function ameliorates AP in mouse models, thus offers potential treatment target of this disease.

In light of the protective action of both Aspirin and Trehalose against AP, we synthesized a new compound that combines both agents, termed Aspirlose. Encouragingly, pre and post‐treatment with this novel compound decreased the elevated levels of amylase and lipase and pancreatic edema characterizing AP in the cerulein model of the disease. In addition, pre‐treatment with Aspirlose in both WT and Hpa‐Tg mice and post‐treatment with Aspirlose in Hpa‐Tg mice, reduced pancreatic inflammatory response. Although Aspirlose was not more effective than combined Aspirin+Trehalose against AP, it has two advantages, first it may improve the compliance of the patients, where taking single drug is preferred over consuming multidrug. Second, Aspirlose may be behave as coated Aspirin, a feature that could minimize its adverse erosive action on the GIT system, although confirming these issues requires additional studies.

In conclusion, our results suggest that Aspirlose may compose a novel therapeutic tool for AP as was evident by reversing the biochemical, inflammatory, and histological perturbations in this disease state.

## AUTHOR CONTRIBUTIONS

**Dalit B. Hamo‐Giladi:** Data curation (equal); formal analysis (equal); investigation (equal); methodology (equal); software (equal); validation (equal); visualization (equal); writing – original draft (equal); writing – review and editing (equal). **Ahmad Fokra:** Data curation (equal); formal analysis (equal); investigation (equal); methodology (equal); writing – original draft (equal). **Edmond Sabo:** Data curation (equal); formal analysis (equal); methodology (equal); software (equal); writing – original draft (equal). **Aviva Kabala:** Data curation (equal); investigation (equal); methodology (equal); visualization (equal). **Irena Minkov:** Formal analysis (equal); methodology (equal); writing – original draft (equal). **Shadi Hamoud:** Formal analysis (equal). **Salim Hadad:** Data curation (equal); formal analysis (equal); investigation (equal); methodology (equal); project administration (equal); writing – original draft (equal). **Zaid Abassi:** Conceptualization (equal); data curation (equal); formal analysis (equal); funding acquisition (equal); methodology (equal); project administration (equal); resources (equal); supervision (equal); validation (equal); visualization (equal); writing – original draft (equal); writing – review and editing (equal). **Iyad Khamaysi:** Conceptualization (equal); data curation (equal); formal analysis (equal); funding acquisition (equal); investigation (lead); methodology (equal); project administration (equal); resources (lead); supervision (lead); validation (lead); visualization (equal); writing – original draft (lead); writing – review and editing (lead).

## CONFLICT OF INTEREST STATEMENT

The authors declare no conflict of interest.

## INSTITUTIONAL REVIEW BOARD STATEMENT

All experiments were approved and performed according to the Technion's guidelines of the Committee for the Supervision of Animal Experiments (IL‐90‐08‐2020), and were consistent with the institutional guidelines and NIH Guidelines for the Care and Use of Laboratory Animals.

## Supporting information


Figure S1.

## Data Availability

The data are available from Z. Abassi upon request.
