# Supplementary figures and images for: Involvement of heparanase in the pathogenesis of acute pancreatitis: Implication of novel therapeutic approaches
Source: J Cell Mol Med. 2024 Sep 9;28(17):e18512. doi: 10.1111/jcmm.18512 (PMC11382361; doi:10.1111/jcmm.18512)

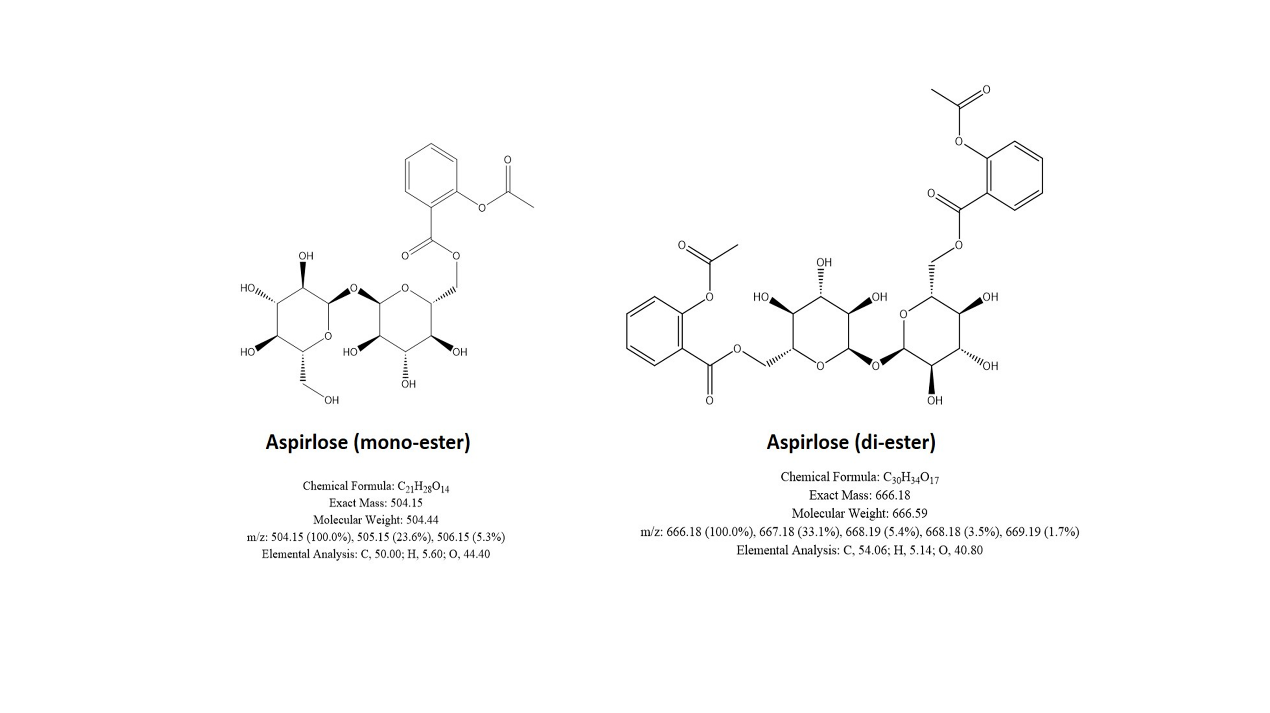


**Supplementary Figure 1**

Supplement: Supplementary file 1 — Figure S1. [file JCMM-28-e18512-s001.docx]
